# On the Structure and Functions of Nervous Tissue
*Principles of Physiology, General and Comparative. By W. B. Carpenter, M.D. 3rd edition. Churchill. 1851.Hand-book of Physiology. By W. S. Kirkes, M.D., assisted by J. Paget, F.R.S. 2nd edition. Churchill. 1851.


**Published:** 1852-01-01

**Authors:** 


### Art. II.—ON THE STRUCTURE AND FUNCTIONS OF NERVOUS TISSUE.*

It is well that we should, from time to time, present to our mind a *résumé* of our knowledge of different organic tissues, with the view of ascertaining the amount of progress we have made in those departments of science specially connected with the province of this journal. A recent edition of Dr. Carpenter's and Dr. Kirke's Physiology enables us to present to our readers a sketch of the state of our knowledge of the structure and functional peculiarities of nervous tissue. We need say nothing in commendation of Dr. Carpenter's labours as a physiologist. The new edition of his work speaks for itself. The labour bestowed upon it must have been immense. Many portions of the work have been entirely re-written. The two volumes before us may be considered fairly to represent the state of physiological science of the present day.

Although endowed with such varied and remarkable properties, the elementary structures through which the nervous tissue manifests its functions are extremely simple, consisting apparently of little else than highly endowed cells for the purpose of originating the nervous force or principle, and an assemblage of tubes or fibres for the transmission of this principle to the parts to be influenced by it. The function of the latter, namely, the fibrous element of the nervous tissue, being thus, as it were, secondary to the former, it might indeed be inferred that the cellular or vesicular element is the only essential constituent of nervous tissue, and there are not wanting arguments in support of this view; but since no animal organization has yet been discovered in which vesicular nervous tissue uncombined with the fibrous element occurs, we are compelled to believe that both these elementary parts of nervous tissue are essential to the production and manifestation of nervous phenomena.

Of these two constituent elements of nervous tissue, namely, the corpuscular and the fibrous, the one is usually collected into masses, or nervous centres, as they are termed, such as the brain, spinal cord, and the various ganglia; while the other, the fibrous element, is disposed in

* Principles of Physiology, General and Comparative. By W. B. Carpenter, M.D. 3rd edition. Churchill. 1851.
Hand-book of Physiology. By W. S. Kirkes, M.D., assisted by J. Paget, F.R.S. 2nd edition. Churchill. 1851.

the form of nervous trunks, issuing from such centres, and proceeding to all parts of the body, which they thus bring into connexion with the nervous centres. In describing the structure of nervous tissue, we will speak first of the fibrous element. This enters largely into the composition of the brain and spinal cord, forming the greater part of the white substance of these nervous centres; it is there, however, mingled and brought into relation with the corpuscular element, while in the nerves which issue from these centres it exists uncombined with corpuscles, and constitutes nearly their entire structure. The fibres or tubules of which this portion of nervous tissue is composed are met with under two distinct forms, characterised by certain peculiarities in size, colour, and structure. Although there is reason to believe that these diversities are more apparent than real, and that there is no more essential difference between the two kinds of fibres than might result from different stages of development of one and the same kind of fibre, yet it will be necessary to speak of each of them separately, and then to state the relation which they seem to bear to each other. Of the two kinds of fibres then, one consists of what are called *tubular* or *white fibres,* the other of *gelatinous* or *grey:* the former comprises most of the fibres found in cerebro-spinal nerves, the latter nearly all those which occur in the sympathetic system. In the nerves both of the cerebro-spinal and the sympathetic systems, however, both sets of fibres are found variously intermingled, so that it cannot be said that either system is composed exclusively of one set of fibres. Since the tubular form of nerve fibre enters so largely into the composition of the cerebro-spinal nerves, its structure can be best determined by an examination of one of these nerves. Each cerebro-spinal nerve-trunk is composed of a number of nerve-fibres arranged in fasciculi or bundles, each of which is surrounded and separated from the rest by a sheath of fine fibro-cellular tissue, while the entire nerve is itself also invested by a similar though coarser fibrous sheath, termed the neurilemma. The use of this investment to the entire nerve and to the individual bundles of which it is composed, is to protect and isolate the elementary fibres of the nerve, and at the same time to furnish them with a net-work of blood-vessels from which they may derive their supplies of nutriment. By separating the several fasciculi of which a nerve is composed, and dividing and subdividing any one of them, we arrive at length at the primitive nerve-fibres or fibrils, which are the essential elements of the fibrous portion of nervous tissue. When carefully examined, each elementary nerve-fibril of this class is found to consist of a delicate homogeneous cylindrical membrane, which forms a kind of isolating sheath or tubule for the proper nerve-substance contained within it. The nature of the substance thus enclosed within the tubular membrane is not yet clearly determined.

When a perfectly fresh cerebro-spinal nerve is examined microscopically, each individual fibril presents the appearance of a fine thread of glass, on account of the contents of the tubules appearing to consist of a clear colourless homogeneous fluid. But very shortly this appearance is lost, and the contents of the tubules undergo changes, which make it probable that, instead of being really homogeneous, they consist of two very different materials. Viewed under these circumstances, a nerve-fibril appears to be constituted first of a layer of soft whitish material, named the *white substance* of Schwann, situated immediately within the membranous tube, and surrounding the other, or second ingredient, which is a clear transparent material, occupying the centre or axis of the fibre, and hence termed the *axis-cylinder*. It is often very difficult to obtain a distinct view of these two individual portions of the nerve-fibre, for the whole substance contained within the tube is very soft, yielding readily to the slightest pressure, and causing the wall of the tube to bulge and become distorted. Moreover, the contents themselves seem to undergo a kind of coagulation, in consequence of which they collect in little masses, which distend the tubular membrane unequally, and cause it to assume a peculiar varicose or beaded appearance, instead of its previous cylindrical form. In spite, however, of these obstacles to a correct determination of the real structure of the primitive nerve-fibrils, it seems to be admitted, by nearly all physiologists of the day, that each fibre is really composed of the distinct portions just named, the one circumferential, the other central. Of these two portions, it is also now generally admitted that the latter, namely, the clear central-axis portion, is the essential component of the nerve-fibre, that on which its true functions depend; while the outer, white, medullary portion, serves only, or chiefly, as an isolator or protector to the important part within, surrounding and defending it in the same way and for the same purpose as both are surrounded by the tubular membrane outside. The principal evidence in favour of this view will be presently adduced.

In size, the nerve-fibrils just described vary considerably, the majority, however, measuring, in the trunk of a nerve, from $\frac{1}{2000}$ to $\frac{1}{3000}$ of an inch in diameter. As a rule, they are much smaller when examined within the nervous centres than they are in the rest of the course, and they are also often noticed to diminish considerably in size previous to their peripheral termination. The fibres of the nerves of special sense, likewise, are smaller than those of the nerves of ordinary sense.

The gray or gelatinous fibres comprise the majority of the fibres found in the trunk and branches of the sympathetic nerve. They differ from those just described in being smaller, finer, of a pale yellowish gray colour, flat instead of cylindrical, without the double contour produced in the tubular fibres by their compound structure, and apparently devoid both of an outer tubular envelope, and of the medullary or white substance of Schwann. They appear, indeed, to consist of little else than a material very similar to that forming the axis-cylinder in the tubular fibres, except that it possesses a finely granular appearance, and is marked by numerous nuclei adhering to its surface. So little general resemblance to the tubular fibres is possessed by these gray or gelatinous fibres, that their title to be regarded as nerve-fibres at all has been disputed. Without pausing, however, to consider this point, since by almost general assent their nervous character is admitted, we will briefly state some of the arguments in favour of the view that these fibres are not, as they at first sight seem to be, different in kind from the tubular fibres, but are merely modifications of them. Admitting as true what has been already stated, that the central part, or axis-cylinder, is the essential component element of the tubular fibre, the general resemblance which the gelatinous fibre presents to this might be deemed conclusive that it possesses the essential structural attributes of the tubular fibre. But stronger evidence of the same kind is afforded by the fact, that at their origin in the nervous centres, and very commonly at their peripheral terminations, the tubular fibres generally lose the white medullary portion which they possess in their course along the nerves, and then present the faint outline and finely granular aspect belonging to a gelatinous fibre. So also in the first development of nerve-fibres as witnessed in the tail of the tadpole, fibres exactly like those of the gelatinous kind may be seen gradually passing into and becoming continuous with proper tubular fibres, which are therefore, probably, only the same structure in a higher phase of development. Again, it is not uncommon to find in the same nerve, mixed with gelatinous and tubular fibres, other fibres, which in general characters are intermediate between the two varieties, thus showing a kind of transition from one kind of fibre to the other, and helping to demonstrate the truth of the view which is now rapidly gaining ground, that there is no real difference between the gelatinous and the tubular fibres; that they both contain the same essential nervous element; and that the small size and homogeneous structure of the gelatinous fibre is due to its being, for some special purpose, less completely developed than the tubular fibre.

The origin, course, and termination of the nerve-fibres will be more advantageously studied after a description of the second component element of nervous tissue—namely, the vesicular or corpuscular structure.

The vesicular structure of nervous tissue consists of nucleated cells, in various stages of development, collected together in masses, and imbedded in a finely granular blastema, which is traversed by blood-vessels and nervous fibrils, the several collections constituting what are termed nervous centres or ganglia. The cells, which are also termed ganglion-corpuscles, or nerve-vesicles, differ greatly in size, some being scarcely larger than a human blood-corpuscle, while others may be one-three-hundredth of an inch in diameter. They are nucleated, very often nucleolated also, and contain a finely-granular or grumous material, in which are occasionally noticed larger and darker particles, giving the corpuscles a peculiar dark brownish appearance. The majority of the corpuscles are spheroidal, though from mutual pressure they often become more or less angular and irregular in shape. Besides these latter irregularities in form, which have long been noticed, other varieties have lately been discovered, which promise to be of great service in helping to a right interpretation of nervous phenomena. The varieties now alluded to are those in which corpuscles present a peculiar caudate or stellate form, in consequence of one or more processes being given off from their surfaces. Usually these processes extend but a short distance from the corpuscle, terminating abruptly, or in a fine point; but occasionally they may be traced much further, and even seen to branch, and not unfrequently one of the branches may be seen to become directly continuous with a nerve-fibre, which thus appears to originate from it, or rather, by means of it, from the corpuscle. The processes which issue from the nerve-corpuscles are moreover tubular, and are filled with granular material, similar to that in the interior of the corpuscle, from which indeed they seem to derive it, for the contents of each appear to be continuous, the processes being so many hollow tubes issuing from and communicating with the interior of the corpuscle. The nerve-fibres, which appear to originate in this manner from the nerve-corpuscles, at first consist simply of the pale granular out-growth of the contents of the corpuscle, but when traced further they may be seen gradually to assume a medullary sheath of white substance, and thus come to resemble an ordinary tubular fibre. This fact has been already partly alluded to as seeming to prove an identity between the tubular and gelatinous fibres.

Although many of the nerve-fibres entering a ganglion or nervous centre are thus found to be directly continuous with the ganglion-corpuscles, yet many other fibres pass through, apparently without forming any such connexion; while there are also found many corpuscles in the various nervous centres, in which no such close relation between them and the nerve-fibres around them exists; the question, therefore, still remains unsettled, whether all nerve-fibres thus originate in ganglion-corpuscles, and if this mode of origin is not universal, whether there is any functional diversity between those fibres which do and those which do not possess this intimate connexion with the ganglion-corpuscles. Dr. Carpenter seems inclined to adopt the view that the cells from which the nerve-fibres seem to spring are those by which they are formed, whilst the globular cells among which they pass are rather the instruments of their functional changes. "This idea," he remarks, "derives confirmation from the researches of Kölliker on the peripheral origin of the nerve-fibres; for he has found that in the tail of the tadpole the nervous plexuses are formed after the same fashion as the capillary net-work, namely, by the inosculation of the prolongations of radiating cells whose centres are at a considerable distance from each other."

Further investigation is, however, necessary before a definite judgment can be pronounced respecting the number and kind of fibres which are thus brought into immediate relation with the ganglion-corpuscles.

This portion of the subject naturally leads to a consideration of the central and peripheral terminations of nerve-fibres, and of certain peculiarities observed in their course from one extremity to the other. The central termination, or perhaps, more correctly, the central origin, of the majority of nerve-fibres, is obviously effected by their becoming connected with ganglion-corpuscles in the manner described. As the fibres enter a nervous centre, they gradually become finer, the outer white substance of the tubular fibres gradually disappears, while the central gelatinous portion becomes continuous with the granular processes arising from the ganglion-corpuscles. Whether all the fibres of both kinds of nerves enter into this close relation with the ganglion-corpuscles is, as already stated, still uncertain, though it seems highly probable that they do.

In their course from their central to their peripheral extremities, the individual fibres of each nerve are supposed to proceed uninterruptedly, each fibre preserving its continuity, without branching or anastomosing, from one extremity to the other.

The phenomena of nervous action are more intelligible according to this view than they would be if it were assumed, as has been recently suggested, that such continuity and singleness of each individual fibre throughout its entire course do not exist. The question, indeed, is not easy of anatomical solution; but since all physiological reasoning is on the side of perfect continuity of fibre, from origin to termination, and no sufficient evidence against this view has yet been advanced, it may still be allowed to stand. Although there is probably no anastomosing or union of the substance of one fibre with that of another in their course along the nerves, yet we frequently observe an intermingling of fasciculi of fibres of different nerves. This is seen in the formation of the various plexuses, and of the nerves which issue from them; the plexuses being formed by the interchange of bundles of fibres from various nerves, while the nerves which emerge therefrom are also made up of fibres derived from several different nerves. The object of such interchange of fibres is obviously to afford the several nerves issuing from the plexus a wider connexion with the nervous centres than they would otherwise have. For example, since the brachial plexus is formed by the intermingling of fibres from the four last cervical and first dorsal nerves, each nervous trunk emerging from this plexus probably contains within it fibres derived from the several parts of the cord intermediate between the origin of the fourth cervical and the first dorsal nerve, and hence the parts supplied by it will have wider relations with the nervous centres, and more extensive sympathies, than they could have if they were supplied by nerves proceeding directly from the spinal cord, and without such intermediate connexion with other nerves.

At their peripheral extremities nerve-fibres terminate in various ways; but in almost every case, previous to their termination, they break up and form delicate networks, called the terminal plexuses. From these plexuses the individual fibres issue, to terminate in the elementary tissue of the parts in which they are placed.

Concerning the mode in which the ultimate fibrils are really disposed of, much doubt still exists; but as far as microscopic investigation at present extends, we are entitled to admit of at least three distinct modes of termination: first, by a kind of looped arrangement, in which the individual fibril, after issuing from a terminal plexus, forms a single narrow loop on the tissue in which it occurs, and then turns back to join the plexus from which it proceeded, or an adjoining one, and thus, probably, pursues its way back to the nervous centres. This mode of termination has been observed in the papillæ of the tongue, the tooth-pulp, the internal ear, and other parts. A second mode of termination has been described as present in serous membranes, in which the nerve-fibres form minute plexuses composed of innumerable delicate fibres. In the third mode of termination the individual fibrils end in free extremities. There is reason to believe that the fibres in the papillæ of the skin, as well as in other parts, terminate in this way; but it is only in the little bodies named Pacinian corpuscles that this mode of termination has been clearly determined. These Pacinian corpuscles are small, oval, elongated bodies, situated on some of the cerebro-spinal and sympathetic nerves, especially on the cutaneous nerves of the hands and feet. Each corpuscle, attached by a narrow pedicle to the extremity of the nerve, is formed of several concentric layers of fine membrane, with intervening spaces filled with fluid. Through the pedicle by which the corpuscle is attached to the nerve, a single nerve-fibril enters it, traversing the concentric layers of membrane and the intervening fluid, and terminates in a knob-like enlargement, or by bifurcation, at or near the distal end of a small central cavity existing in the interior of the corpuscle. As the fibril enters the corpuscle it gradually loses its outer investing portion, and while traversing the central cavity is very small and delicate, and appears to consist of little else than its central gelatinous portion. There are certain peculiarities often observed in the mode of termination of the nerve-fibre within the corpuscle, but into these it is not necessary here to enter, the account just given being applicable to the majority of the corpuscles.

The chemical composition of nervous tissue demands a few remarks. Owing to the presence of blood and capillary blood-vessels, and probably other accessory tissues in nervous matter, it is scarcely possible to submit it to an exact chemical analysis. But the same difficulty exists in the case of most other tissues, and need scarcely be considered in the results of the ordinary analyses to which nervous tissue has been submitted, and which show it to consist of albumen, fatty matter, and salts, combined with a very large proportion of water. The large quantity of water, amounting to from four-fifths to seven-eighths of the whole tissue, is very remarkable; and, as observed by Dr. Carpenter, is especially interesting when considered in relation to the fact that the "vital activity of a tissue is usually greater, as the proportion of its solid to its fluid contents is less;" for there is no tissue whose vital energies are so active as those of the nervous tissue. According to Frémy, the cerebral substance, which may be taken to represent nerve-substance in general, consists of 80 per cent. of water, 7 of albumen, and 5 of fatty constituents. The albuminous ingredient requires no comment here. The fatty principles are remarkable from the fact that two of them, which are acid compounds, contain a large proportion of phosphorus. The total amount of phosphorus thus existing in the brain is very considerable, being from 8 to 18 parts in 1000 of the whole mass, or from one-twentieth to one-thirtieth of the entire solid matter. This important ingredient in the nervous tissue appears to be continually given off during the change and disintegration which ensue in the nervous as well as other tissues in the discharge of their ordinary functions.

It is now a well-established fact, that in every act of an organized structure, there is a corresponding change or death of a certain amount of the acting tissue, the act indeed being the manifestation of the chemical change, and the index of its amount. The elements of the parts thus changed or decayed assume new forms and combinations, leave the tissues to which they are no longer of use, and often re-appear in a discernible form in some other part of the body, affording thus a proof of the disintegration of a given tissue, and a tolerable estimate of the amount of waste undergone. We observe this especially in the muscular tissue, the amount of the disintegration of which is pretty accurately determined by the quantity of urea into which the elements of the wasted tissue resolve themselves, found in the urine. So, too, in the nervous system, a tolerably correct estimate of its activity can be formed, by determining the amount of alkaline phosphates found in the urine : for the phosphorus set free by the disintegration of nervous tissue during the discharge of its active functions, unites, in the form of an acid, with the alkaline bases in the blood, and is thence separated at the kidneys, and discharged with the urine. In this manner, may be explained the alkaline urine, depending on excess of alkaline phosphates, after undue exercise of the mind, which is necessarily attended with an unusual waste of the cerebral tissue.

In forming an estimate of the amount of disintegrated cerebral tissue, by the quantity of phosphates present in the urine, the alkaline phosphates alone have to be considered, for the quantity of earthy phosphates present in the urine have been shown by Dr. B. Jones to be dependent on the quantity taken in as food, and not on the decay of nervous tissue.

Having offered this brief outline of the structural and chemical peculiarities of nervous tissue, we proceed to give a general sketch of its functions. Nervous tissues being composed, as already shown, of two distinct portions,—a vesicular, designed to originate nervous impulses and to take cognizance of impression conveyed to it, and a fibrous, designed to transmit the nervous influence to and from the vesicular portion,—it follows, that in considering the general functions of the nervous tissue, we must deal separately with each of these two elementary parts, of which the tissue is composed. But it may be as well to state generally, in the first place, what is the special purpose and office of the nervous tissue considered as a whole : and in doing this, no better words can be used than those of Dr. Carpenter, who says,—

"The functions of the nervous system are two-fold : first, to bring the conscious mind (using that term in its most extended sense, to denote the psychical endowments of animals in general) into relation with the external world—by informing it, through the medium of the organs of sensation, of the changes which material objects undergo : and by enabling it to re-act upon these through its motor apparatus : and also to connect and harmonize different actions in the same individual, without necessarily exciting any mental operation. These two sets of purposes, however, are fulfilled by a mechanism of the same kind. An 'impression' made upon some part of the general surface of the body, or upon a special organ of sense, is received by the nerve-fibres, which originate in it, and is propagated by them to some part of the central ganglionic apparatus. If the impression reach the portion termed the *sensorium*, (which is always seated in the *head*, where this can be distinguished,) it then affects the *consciousness* of the animal, and becomes a *sensation*, provided the sensorium be in a con-

ARMY MEDICAL LIBRARY
WASHINGTON, D. C. U. S. A.

dition of activity. The sensation thus produced may give rise to ideas, and these to reasoning processes, which may terminate in an act of the will; and this playing, as it were, upon the nervous apparatus, produces a change in the condition of that portion of the nervous centres from which the motor nerves arise, whence it is propagated by these nerves to certain muscles, and excites them to contraction. But the sensation may, in itself, more directly excite a motor action, without any reasoning process or voluntary effort: and the movement is then said to be 'automatic.' Farther, even if the impression do not reach the sensorium, it may still excite a motor action through some other nervous centres: and the movement thus produced is 'automatic' like the preceding, from which it differs, in not being prompted by a sensation, but in resulting from the conveyance of the simple impression to a portion of the nervous centres capable of originating a respondent action."

Such being the general functions of the nervous system, we will proceed to consider, rather more in detail, the share taken by the two elementary portions of the tissue, through the influence of which these functions are discharged. That the vesicular portion of nervous tissue is the seat of all the active powers of the nervous system, while the fibrous portion merely serves to conduct nervous impressions to or from them, may be proved in various ways, but, perhaps, most readily by an experiment of the following kind. If a nerve, which is but a collection of the fibrous elements, be divided at any part of its course, all the parts supplied by the portion thus separated from the nervous centres, will be paralysed both in motion and sensation, while the parts supplied by the portion still in connexion with the nervous centre will retain their sensation and power of motion. Moreover, irritation of that portion of the divided nerve in connexion with the nervous centre, excites sensations which are referred to the parts supplied by the other portion of the divided nerve, showing that it is at the nervous centres alone that impressions are perceived as sensations; on the other hand, irritation of the separated portion of the divided nerve is followed by motion in the muscular parts supplied by this portion, showing that the nerve retains its power of conducting impressions though it is unable to originate them.

In the discharge of their respective shares of the functions of the nervous system, the fibrous and vesicular elements appear to be governed by certain laws into which it may now be desirable briefly to inquire. For this purpose, we will follow the account given in Dr. Kirkes's 'Handbook of Physiology,' and will commence with the fibrous portion of the nervous tissue. Speaking generally, nerve-fibres may be said to convey impressions in two directions; first, they convey to the centres impressions made on their peripheral extremities; secondly, they transmit impressions from the brain and other nervous centres, to the parts to which they are distributed, these latter impressions being of, at least, two kinds, namely, those which excite to muscular action, and those which influence the nutrition, secretion, and other organic functions of a part. As far as we, at present, know, the same fibre cannot convey impressions in opposite directions, neither does it seem to be able to convey impressions of different kinds, even in the same direction. Hence, there are, at least, two distinct sets of fibres provided, the one to transmit impressions from the nervous centres, and hence called centrifugal or efferent fibres, the other to convey impressions towards the centres, and hence called centripetal or afferent.

The former set comprises the nerves of motion, the latter the nerves of special and ordinary sensation : the nervous influence by which the organic functions of nutrition, secretion, and the like, are governed, being supposed to be conveyed along both sets of fibres. Although the fibres thus differ in function, yet there is no obvious difference in structure, neither does the tissue to which they are distributed, determine the kind and direction of the impression they convey, for the muscular tissue is endowed with both motor and sensitive properties.

In order that nerve-fibres may act and convey impressions, they must be stimulated, since they possess no power of originating impressions in themselves. Under ordinary circumstances, nerves of sensation are stimulated by external objects acting upon their extremities; and the nerves of motion by the will, or by some force generated in the nervous centres. But almost all things that can disturb the nerves from their passive state act as stimuli, and in this way all chemical, mechanical, or electric irritation will excite nearly the same effect as the natural stimuli, provided they are not so violent as to destroy or seriously injure the fibre to which they are applied.

Some of the laws and conditions of action observed in nerve-fibres apply to both sensitive and motor nerves, while some are peculiar to nerves of motion, others to nerves of sensation. Of the laws common to both fibres we may mention that an impression made on any fibre is transmitted along it simply and uninterruptedly without being diffused among any of the fibres lying near it. This is in accordance with the view that each fibre is simple and continuous from its central to its peripheral extremity, while the complete isolation afforded by the tubular sheath around it is probably the reason why the impression is not imparted to the surrounding fibres, the sheath acting like the isolating covering to an electric wire. Again, the rate at which nervous force travels is immeasurably rapid; possibly some interval does elapse in the transit of an impression, but it is too small to be appreciated.

Another law is, that the same fibre, as has been already stated, cannot convey more than one kind of impression. Thus a motor nerve cannot convey sensitive impressions, neither can a sensitive nerve convey motor impressions : so too, a nerve of special sense can only convey those impressions which give rise to the sense peculiar to the organ it supplies. The only apparent departure from this law has been already stated, namely, that the impressions governing the organic functions of nutrition and secretion seem to be capable of passing along both sensitive and motor filaments.

With respect to the laws peculiar to the nerves of sensation, we find first, that impressions are conveyed only in the direction from their periphery towards the nervous centres in which they arise. Thus, if a sensitive nerve is divided, and irritation is applied to the portion still connected with the nervous centre, sensation is perceived, but no effect ensues while the other portion of the divided nerve is irritated. Secondly, an impression made upon any part of the course of a sensitive nerve is perceived both there and at all the parts to which the fibres of the irritated portion of the nerve are distributed ; the explanation being, that the mind always refers the impression produced by irritation of a sensitive nerve to the peripheral extremities of the fibres of such nerve. In this way is explained the fact that when part of a limb has been removed, irritation of the nerve of the stump produces sensations referred to the lost part: the same is sometimes noticed after division of a nerve for neuralgia. The only law specially relating to motor nerve-fibre is the converse of one already mentioned as belonging to sensitive fibres, namely, that motor impressions are conveyed only in the direction from the centre towards the circumference. Thus, if the distal end of a motor nerve be irritated, contractions of the muscles supplied by its branches ensue, while no result follows the application of the irritant to the portion of the nerve still in connexion with the nervous centre.

The vesicular nerve-substance is collected, as already described, into masses denominated nervous centres ; as, for example, the brain, spinal cord, and the several ganglia. In speaking, therefore, of the functions of the vesicular portion of nervous tissue, we shall have to consider the functions of nervous centres generally. The remarks already offered will have shown that the office of a nervous centre is of a twofold nature—namely, to take cognizance of impressions brought to it by the nervous fibres, and to originate (either in consequence, or independent of such impressions) impulses which are transmitted to various parts of the body, for the purpose of determining and governing their functions. The instances in which nervous centres can be said to originate impulses, independently of being excited thereto, either by the mandates of the will, or by impressions conveyed to them from without, are however very few. For in the majority of cases, the impulses are consequent on an act of volition, or are called into existence by the transmission of an impression to the centres, through centripetal fibres. The nervous force which excites the rhythmical action of the heart may, however, be said to be issued spontaneously from the nervous ganglia within this organ; and the spinal cord may be said to generate spontaneously the force requisite to keep the sphincter ani in contraction; but beyond these, and a few other similar examples, the nervous force which issues from a nervous centre is called into existence by some applied stimulus, and does not originate in a spontaneous action of such centre. For example, the nervous influence requisite to make a muscle contract is, in most cases, excited either by an act of will, or by some impression conveyed to the nervous centre by a centripetal nerve. Although, therefore, we are justified in considering a nervous centre as really an originator of nervous force, yet we must do so with certain qualifications, and admit that some external stimulus is in most cases necessary, in order that the nervous centre may develope the nervous influence which is to proceed from it. In developing this influence in consequence of a stimulus conveyed to them, and in disposing of impressions so received, nervous centres manifest various peculiarities, which require to be noticed. Thus, an impression conveyed by a sensitive nerve may be *conducted* through the nervous centre which it first reaches to some other adjacent or even distant centres. For example, the stimulus afforded by the presence of food in the intestinal canal, is conveyed along the nerves distributed over the intestinal mucous membrane, to adjacent sympathetic ganglia, and usually excites merely muscular contraction of the coats of that portion of the intestine in which the food is contained. But if irritant substances are mixed with the food, the impression conveyed to the ganglia is stronger, and is then conducted through them to other adjacent ganglia, whereby larger portions of the intestinal walls are excited to contraction; or it may be further conducted to the spinal centres, and lead to the production of spasmodic contraction of the abdominal, or other muscles; or lastly, it may even be conducted to the brain, and give rise to the sensation of pain. Instead of, or even as well as, being conducted, impressions reaching nervous centres may also be either *transferred*, or *diffused*, or *reflected*. An example of the first mode of disposal is afforded in the pain occasionally felt in the knee in cases of disease of the hip-joint. The impression conveyed to the nervous centres by the sensitive nerves supplying the diseased joint, is, at such centres, transferred to the central extremities of the nerves of the knee; and thus the mind is led to refer the morbid impression to the part from which these nerves ordinarily convey impressions. An example of the *diffusion* or radiation of impressions received at a nervous centre, is furnished in the well-known fact that continued tooth-ache is often followed by pain in the adjoining teeth, or even in all the surrounding parts of the face. The explanation of this seems to be, that the morbid impression conveyed to the nervous centre, is thence—not merely transferred, as in the last case, but diffused and radiated so as to influence the various other fibres entering the same centre—and thus to excite sensations referred by the mind to the parts from which these secondarily affected nerve-fibres proceed.

Besides being thus communicated from one sensitive fibre to others of the same kind, as in the instances last named, a morbid impression may also be transferred or *reflected* from a sensitive to one or more motor nerves, and thus involuntary muscular action may be induced. In this manner arise all those singular movements denominated *reflex*. As an example may be mentioned the contraction of the iris, consequent on the impingement of a ray of light on the sentient surface of the retina. The stimulus thus applied to the optic nerve is conveyed to the brain, and thence reflected to the central end of the third pair of cerebral nerves, along which motor impulses are transmitted to the iris, and induce its contraction. Numerous other similar examples of reflex action might be mentioned, but the above will serve as an illustration of the class; and the several conditions which exist in it are essential in every other instance of reflected movement. These essential conditions are three: namely, first a centripetal nerve-fibre to convey an impression to the nervous centre; secondly, a nervous centre to which this impression may be conveyed, and in which it may be reflected; and thirdly, a motor or centrifugal nerve, on which this impression may be reflected, and by which it may be transmitted to the contracting tissue. No proper reflex movement can take place if any one of these three conditions is wanting.

There are certain peculiarities in reflex movements which require a few remarks. For example—all reflex movements are essentially involuntary; being quite independent of the will, though admitting of a certain amount of control by a voluntary effort. This is seen in the movements of respiration, which are of the reflex kind; they continue during sleep or coma, though in the wakeful and conscious state they may be variously modified by an effort of the will. All such movements again have an obvious purpose, designed for the welfare of the body; as, for example, the movement of the heart, of the digestive organs; the closure of the eyelids and pupils to exclude excess of light; the spasmodic closure of the glottis against the ingress of foreign substances; and others of a like character. Such movements also may be continued for any length of time without producing weariness; this is well seen in the movements of the heart and of the respiratory muscles.

The outline which has here been offered of the functions of nervous tissue applies to the nervous system generally. There are, however, many peculiarities displayed by different parts of the nervous system in the discharge of their functions, such as those of the nerves of special sense, and of the sympathetic system; but to enter fully into these would enlarge the present article much beyond its proper limits, even if it were not foreign to its special purpose. A few words, however, must be said respecting the mode in which the nervous tissue acts—*i. e.*, the power or force by which it is enabled instantaneously to transmit its influences from one part of the body to another; especially since this portion of the subject is treated at some length by Dr. Carpenter, in the work which has furnished the basis of the present article. As far as concerns our actual knowledge of the nature and mode of action of the nervous force, we may indeed be said to be entirely ignorant; and in this state of ignorance we are perhaps likely ever to remain. But the great similarity evidently subsisting between nervous and electric phenomena has naturally led to much speculation as to the possible identity of the two forces. Dr. Carpenter has well discussed this subject, and thus briefly expresses what is probably the best mode of viewing the subject :—

"Notwithstanding the strong *analogy* which exists between these two powers, we are *not* warranted in regarding them as *identical;* and we should probably best express their true affinity, by saying that these are so closely 'correlated,' that each may be the means of exciting the other."

Thus, the nervous force is evidently capable of being excited by electricity; for if this latter force is applied to a motor nerve, contraction of the muscles supplied by it ensues; and if applied to a sensitive nerve, whether of common or ordinary sensation, the mind perceives a sensation exactly similar to that conveyed by the nerve when excited by its ordinary and natural stimulus. That the converse is also true—namely, that electricity may be excited by the nervous force—is shown in the phenomena displayed by electric fishes, the operation of whose electric organs evidently depends upon their connexion with the nervous centres, and varies in intensity according to the amount of that connexion.

Equally with electricity, we find that heat, light, motion, and chemical affinity, possess the power of exciting nervous action; while, conversely, the nervous force seems to be able to develope or modify each of the above-named forces: hence, it must be considered to be as much correlated with them as it is with the electric force, or as each of these several forces are with it and with each other.

